# Supplementary material for: Re-thinking “non-response” to wasting treatment: Exploratory analysis from 14 studies
Source: PLOS Glob Public Health. 2025 Feb 12;5(2):e0003741. doi: 10.1371/journal.pgph.0003741 (PMC11819528; doi:10.1371/journal.pgph.0003741)
Supplement: S3 Table — (DOCX) [file pgph.0003741.s003.docx]

## **S3 Table:** Individual and nutritional programme characteristics between recovered and non-responders, at enrolment, exit and over follow-up.

|  | **N** | **levels** | **Recovered^1^**  **n =13,727** | **Non-responder^1^**  **n=2,243** | **Total**  **15,970** | **P*** |  |  |  |
| --- | --- | --- | --- | --- | --- | --- | --- | --- | --- |
| Sex | 15,970 | Male | 5,971 (43) | 943 (42) | 6,914 (43) | 0.205 |  |  |  |
|  |  | female | 7,756 (57) | 1,300 (58) | 9,056 (57) |  |  |  |  |
| Age, months | 15,970 | Median (IQR) | 17.2 (11.0 to 27.5) | 15.0 (9.0 to 28.0) | 17.0 (10.6 to 27.9) | <0.001 |  |  |  |
|  |  | 6-23 | 9,067 (66) | 1,501 (67) | 10,568 (66) | 0.435 |  |  |  |
|  |  | 24-59 | 4,660 (34) | 742 (33) | 5,402 (34) |  |  |  |  |
| HIV status | 12,395 | Negative | 10,578 (77) | 1,756 (78) | 12,334 (77) | 0.001 |  |  |  |
|  |  | positive | 43 (0) | 18 (1) | 61 (0) |  |  |  |  |
|  |  | (Missing) | 3,106 (23) | 469 (21) | 3,575 (22) |  |  |  |  |
| Acute malnutrition, baseline | 15,970 | SAM^2^ | 5,546 (40) | 1,545 (69) | 7,091 (44) | <0.001 |  |  |  |
| **^(WHO definition)^** |  | MAM^2^ | 8,181 (60) | 698 (31) | 8,879 (56) |  |  |  |  |
| Oedema, baseline | 15,970 |  | 0 | 0 | 0 |  |  |  |  |
| Oedema, endline | 13,105 | No oedema | 1 (0) | 19 (1) | 20 (0) | <0.001 |  |  |  |
|  |  | (Missing) | 2,363 (17) | 502 (22) | 2,865 (18) |  |  |  |  |
| Weight, kg at baseline | 15,966 | Median (IQR) | 7.1 (6.3 to 8.3) | 6.5 (5.7 to 8.0) | 7.0 (6.2 to 8.3) | <0.001 |  |  |  |
| Weight, kg at endline | 15,949 | Median (IQR) | 8.0 (7.0 to 9.2) | 7.2 (6.4 to 8.9) | 7.9 (6.9 to 9.1) | <0.001 |  |  |  |
| Absolute weight gain, kg | 15,945 | Median (IQR) | 0.8 (0.5 to 1.2) | 0.9 (0.4 to 1.1) | 0.8 (0.5 to 1.1) | 0.016 |  |  |  |
|  |  | (Missing) | 9 (<1) | 16 (1) | 25 (<1) |  |  |  |  |
| Daily weight gain, g/kg/day | 15,797 | Median (IQR) | 2.4 (1.5 to 4.0) | 1.1 (0.6 to 1.7) | 2.2 (1.3 to 3.7) | <0.001 |  |  |  |
|  |  | (Missing) | 155 (1) | 18 (1) | 173 (1) |  |  |  |  |
| Height/length, cm at baseline | 14,685 | Median (IQR) | 72.5 (67.5 to 80.0) | 71.0 (65.9 to 82.0) | 72.4 (67.0 to 80.0) | <0.001 |  |  |  |
| Height/length, cm at endline | 14,181 | Median (IQR) | 73.5 (68.4 to 80.9) | 73.9 (68.2 to 84.0) | 73.5 (68.4 to 81.0) | 0.027 |  |  |  |
| Absolute height/length gain, cm | 14,064 | Median (IQR) | 0.5 (0.0 to 1.1) | 1.0 (0.2 to 2.7) | 0.5 (0.0 to 1.3) | <0.001 |  |  |  |
| MUAC, mm at baseline | 15,953 | Median (IQR) | 120 (114 to 122) | 114 (110 to 120) | 119 (113 to 122) | <0.001 |  |  |  |
|  |  | <115 | 4,048 (29) | 1,196 (53) | 5,244 (33) | <0.001 |  |  |  |
|  |  | <125 | 12,853 (94) | 2,206 (98) | 15,059 (94) | <0.001 |  |  |  |
|  |  | (Missing) | 17 (0) | 0 (0) | 17 (0) |  |  |  |  |
| MUAC, mm at endline | 15,938 | Median (IQR) | 127 (124 to 130) | 120 (117 to 124) | 126 (120 to 130) | <0.001 |  |  |  |
|  |  | <115 | 464 (3) | 357 (16) | 821 (5) | <0.001 |  |  |  |
|  |  | <125 | 3,808 (28) | 1,762 (79) | 5,570 (35) | <0.001 |  |  |  |
|  |  | (Missing) | 20 (0) | 12 (1) | 32 (0) |  |  |  |  |
| Absolute MUAC gain, mm | 15,922 | Median (IQR) | 8 (4 to 12) | 7 (1 to 10) | 8 (4 to 12) | <0.001 |  |  |  |
|  |  | (Missing) | 36 (<1) | 12 (1) | 48 (<1) |  |  |  |  |
| WHZ, baseline | 14,678 | Median (IQR) | -2.4 (-3.0 to -1.9) | -3.0 (-3.5 to -2.3) | -2.5 (-3.1 to -2.0) | <0.001 |  |  |  |
|  |  | <-3 | 3,046 (22) | 956 (43) | 4,002 (25) | <0.001 |  |  |  |
|  |  | <-2 | 9,013 (66) | 1,700 (76) | 10,713 (67) | <0.001 |  |  |  |
|  |  | (Missing) | 1072 (8) | 220 (10) | 1,292 (8) |  |  |  |  |
| WHZ, endline | 13,853 | Median (IQR) | -1.4 (-1.8 to -0.9) | -2.2 (-2.8 to -1.6) | -1.5 (-1.9 to -0.9) | <0.001 |  |  |  |
|  |  | <-3 | 139 (1) | 360 (16) | 499 (3) | <0.001 |  |  |  |
|  |  | <-2 | 1,914 (14) | 1,093 (49) | 3,007 (19) | <0.001 |  |  |  |
|  |  | (Missing) | 1,688 (12) | 429 (19) | 2,117 (13) |  |  |  |  |
| Absolute WHZ gain | 13,736 | Median (IQR) | 1.0 (0.5 to 1.6) | 0.8 (0.0 to 1.3) | 1.0 (0.5 to 1.6) | <0.001 |  |  |  |
|  |  | (Missing) | 1,771 (13) | 463 (21) | 2234 (14) |  |  |  |  |
| WAZ, baseline | 15,966 | Median (IQR) | -3.2 (-3.9 to -2.6) | -3.6 (-4.3 to -2.9) | -3.2 (-3.9 to -2.6) | <0.001 |  |  |  |
|  |  | <-3 | 8,156 (59) | 1,564 (70) | 9,720 (61) | <0.001 |  |  |  |
|  |  | <-2 | 12,356 (90) | 2,092 (93) | 14,448 (90) | <0.001 |  |  |  |
|  |  | (Missing) | 0 (0) | 4 (0) | 4 (0) |  |  |  |  |
| WAZ, endline | 15,822 | Median (IQR) | -2.3 (-3.0 to -1.7) | -2.7 (-3.5 to -1.9) | -2.4 (-3.0 to -1.7) | <0.001 |  |  |  |
|  |  | <-3 | 3,205 (23) | 881 (39) | 4,086 (26) | <0.001 |  |  |  |
|  |  | <-2 | 8,570 (62) | 1,555 (69) | 10,125 (63) | <0.001 |  |  |  |
|  |  | (Missing) | 100 (1) | 48 (2) | 148 (1) |  |  |  |  |
| Absolute WAZ gain | 15,818 | Median (IQR) | 0.8 (0.5 to 1.2) | 0.8 (0.5 to 1.3) | 0.8 (0.5 to 1.2) | 0.536 |  |  |  |
|  |  | (Missing) | 100 (1) | 52 (2) | 152 (1) |  |  |  |  |
| MUAC Z at baseline | 15,953 | Median (IQR) | -2.8 (-3.4 to -2.3) | -3.3 (-3.8 to -2.7) | -2.9 (-3.5 to -2.3) | <0.001 |  |  |  |
|  |  | <-3 | 5,826 (42) | 1,415 (63) | 7,241 (45) | <0.001 |  |  |  |
|  |  | <-2 | 11,775 (86) | 2,126 (95) | 13,901 (87) | <0.001 |  |  |  |
|  |  | (Missing) | 17 (0) | 0 (0) | 17 (0) |  |  |  |  |
| MUAC Z at endline | 15,758 | Median (IQR) | -1.9 (-2.5 to -1.5) | -2.6 (-3.2 to -2.1) | -2.0 (-2.6 to -1.5) | <0.001 |  |  |  |
|  |  | <-3 | 1,621 (12) | 720 (32) | 2,341 (15) | <0.001 |  |  |  |
|  |  | <-2 | 6,221 (45) | 1,717 (77) | 7,938 (50) | <0.001 |  |  |  |
|  |  | (Missing) | 185 (1) | 27 (1) | 212 (1) |  |  |  |  |
| Absolute MUAC Z gain | 15,742 | Median (IQR) | 0.8 (0.4 to 1.2) | 0.7 (0.1 to 1.1) | 0.8 (0.4 to 1.2) | <0.001 |  |  |  |
| HAZ, baseline | 14,650 | Median (IQR) | -2.7 (-3.6 to -1.8) | -2.6 (-3.7 to -1.6) | -2.7 (-3.6 to -1.7) | 0.109 |  |  |  |
|  |  | <-3 | 5,049 (37) | 805 (36) | 5,854 (37) | <0.001 |  |  |  |
|  |  | <-2 | 8,705 (63) | 1,307 (58) | 10,012 (63) | <0.001 |  |  |  |
|  |  | (Missing) | 1095 (8) | 225 (10) | 1,320 (8) |  |  |  |  |
| HAZ, endline | 13,923 | Median (IQR) | -2.4 (-3.4 to -1.5) | -2.1 (-3.2 to -1.0) | -2.4 (-3.4 to -1.4) | <0.001 |  |  |  |
|  |  | <-3 | 4,122 (30) | 538 (24) | 4,660 (29) | <0.001 |  |  |  |
|  |  | <-2 | 7,540 (55) | 950 (42) | 8,490 (53) | <0.001 |  |  |  |
|  |  | (Missing) | 1,639 (12) | 408 (18) | 2,047 (13) |  |  |  |  |
| Absolute HAZ gain | 13,805 | Median (IQR) | 0.2 (0.0 to 0.4) | 0.4 (0.0 to 1.0) | 0.2 (0.0 to 0.4) | <0.001 |  |  |  |
| Concurrently wasted and stunted^3^, | 14,657 | Yes | 6,182 (45) | 1,078 (48) | 7,260 (45) | <0.001 |  |  |  |
| baseline |  | (Missing) | 1089 (8) | 224 (10) | 1,313 (8) |  |  |  |  |
| Concurrently wasted and stunted, | 13,893 | Yes | 1103 (8) | 556 (25) | 1,659 (10) | <0.001 |  |  |  |
| endline |  | (Missing) | 1,654 (12) | 423 (19) | 2,077 (13) |  |  |  |  |
| Number of visits in the nutritional programme | 15,970 | Median (IQR) | 5.0 (3.0 to 7.0) | 8.0 (7.0 to 13.0) | 5.0 (3.0 to 8.0) | <0.001 |  |  |  |
| Length of stay in the nutritional programme, weeks | 15,970 | Median (IQR) | 6.0 (4.0 to 10.0) | 14.6 (12.6 to 17.3) | 7.3 (4.0 to 12.0) | <0.001 |  |  |  |
|  |  | 1-4 | 5,114 (37) | 34 (2) | 5,148 (32) | <0.001 |  |  |  |
|  |  | 5-7 | 3,020 (22) | 71 (3) | 3,091 (19) |  |  |  |  |
|  |  | 8-12 | 3,545 (26) | 465 (21) | 4,010 (25) |  |  |  |  |
|  |  | 13-16 | 1,715 (12) | 1,000 (45) | 2,715 (17) |  |  |  |  |
|  |  | 17 and more | 333 (2) | 673 (30) | 1006 (6) |  |  |  |  |
| Morbidity^4^ | 13,370 | None | 4,783 (35) | 581 (26) | 5,364 (34) | <0.001 |  |  |  |
|  |  | At least one | 6,889 (50) | 1,117 (50) | 8,006 (50) |  |  |  |  |
|  |  | (Missing) | 2,055 (15) | 545 (24) | 2,600 (16) |  |  |  |  |
| Hospitalisation | 15,970 | Hospitalised | 408 (3) | 194 (9) | 602 (4) | <0.001 |  |  |  |
|  |  | No hospitalised | 2,698 (20) | 275 (12) | 2,973 (19) |  |  |  |  |
|  |  | (Missing) | 10,621 (77) | 1,774 (79) | 12,395 (78) |  |  |  |  |
| SAM^2^, endline | 11,191 |  | 596 (4) | 573 (26) | 1,169 (7) | <0.001 |  |  |  |
|  |  | (Missing) | 3,963 (29) | 816 (36) | 4,779 (30) |  |  |  |  |
| MAM^2^, endline | 12,367 |  | 3,284 (24) | 1,247 (56) | 4,531 (28) | <0.001 |  |  |  |
|  |  | (Missing) | 3,057 (22) | 546 (24) | 3,603 (23) |  |  |  |  |

Data are n (%) or median (IQR). IQR=interquartile range. MUAC=mid-upper-arm circumference. WHZ=weight for-height/length z score. WAZ=weight-for-age z score. HAZ=height/length-for-age z score.

*Univariate analysis comparing recovered vs. non-responder groups. Chi-squared tests for binary and categorical variables; Kruskal-Wallis for continuous variables.

^1^ These categories refer to discharge criteria from treatment used in the individual studies and differ between studies; further information in Supplementary Table S1.

^2^ WHO definition of severe acute malnutrition (SAM) is defined as MUAC <115 mm or WHZ <–3 or oedema. Children with oedema or with missing data on oedema are excluded from this analysis. Moderate acute malnutrition (MAM) is defined as MUAC ≥115 mm and < 124 mm or WHZ ≥-3 and < -2, with no oedema.

^3^ Concurrently wasted and stunted defined as HAZ <-2 and WHZ <-2.

^4^ Morbidity includes caregiver-reported diarrhoea, cough, fever, vomiting and/or rash, malaria at inclusion or during follow-up.
