# Supplementary figures and images for: Re-thinking “non-response” to wasting treatment: Exploratory analysis from 14 studies
Source: PLOS Glob Public Health. 2025 Feb 12;5(2):e0003741. doi: 10.1371/journal.pgph.0003741 (PMC11819528; doi:10.1371/journal.pgph.0003741)

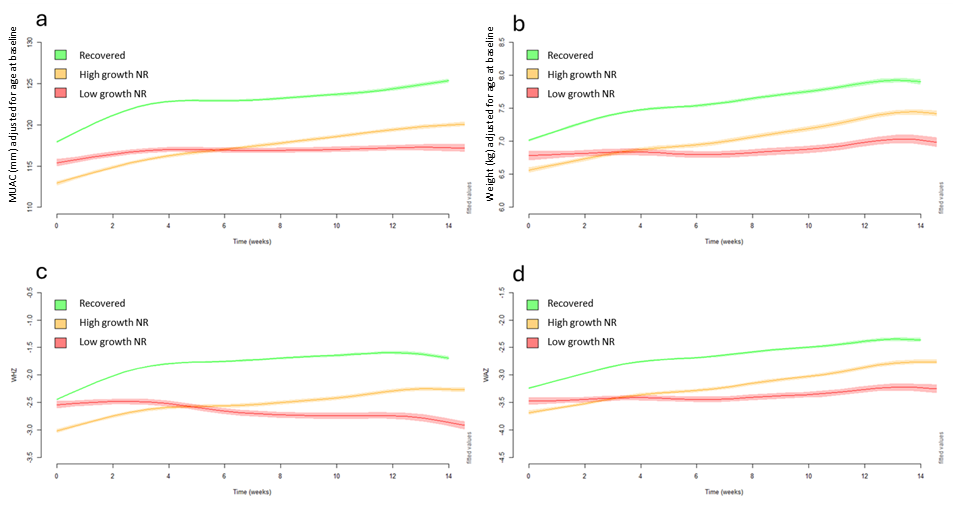

Supplement: S1 Fig — MUAC, mid-upper-arm circumference; WAZ, weight-for-age z-score; WHZ, weight-for-height z-score. (TIF) [file pgph.0003741.s004.tif]

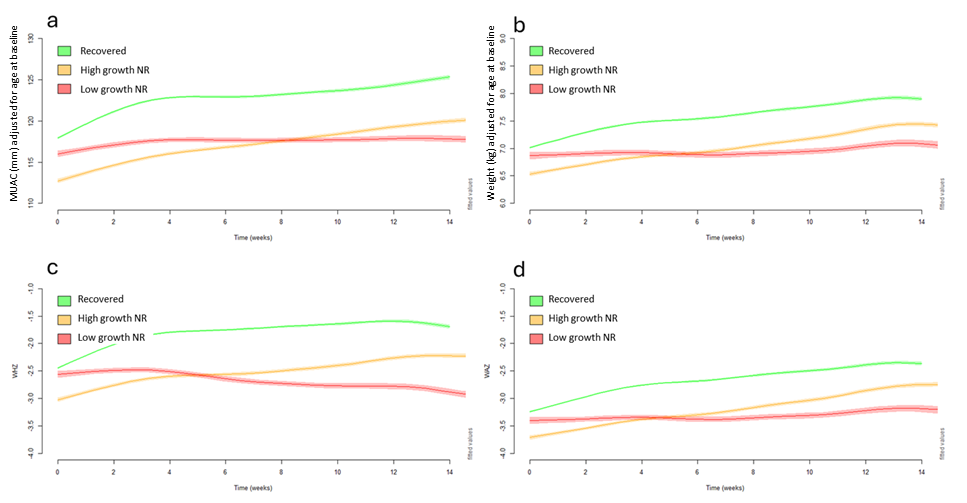

Supplement: S2 Fig — MUAC, mid-upper-arm circumference; WAZ, weight-for-age z-score; WHZ, weight-for-height z-score. (TIF) [file pgph.0003741.s005.tif]

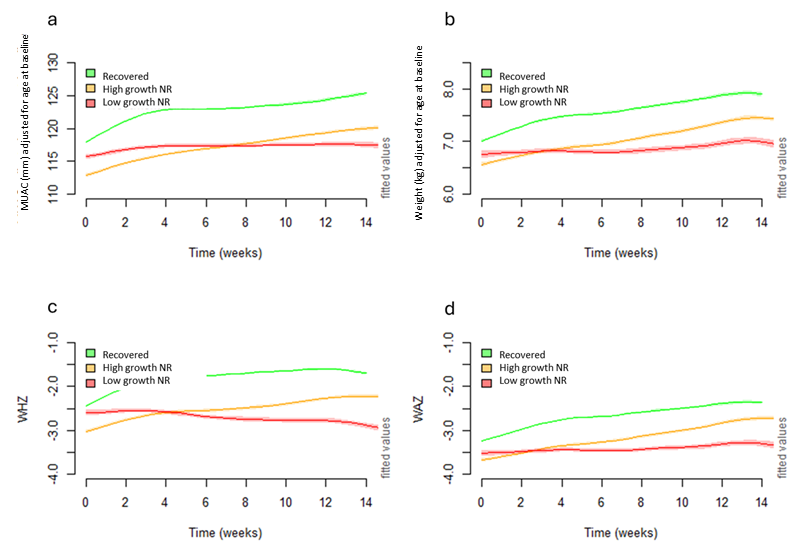

Supplement: S3 Fig — MUAC, mid-upper-arm circumference; WAZ, weight-for-age z-score; WHZ, weight-for-height z-score. (TIF) [file pgph.0003741.s006.tif]

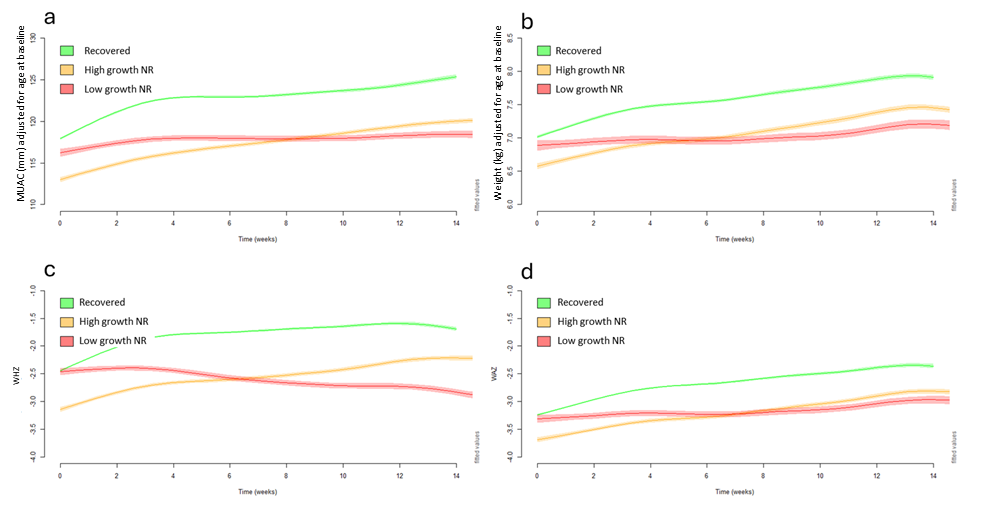

Supplement: S4 Fig — MUAC, mid-upper-arm circumference; WAZ, weight-for-age z-score; WHZ, weight-for-height z-score. (TIF) [file pgph.0003741.s007.tif]

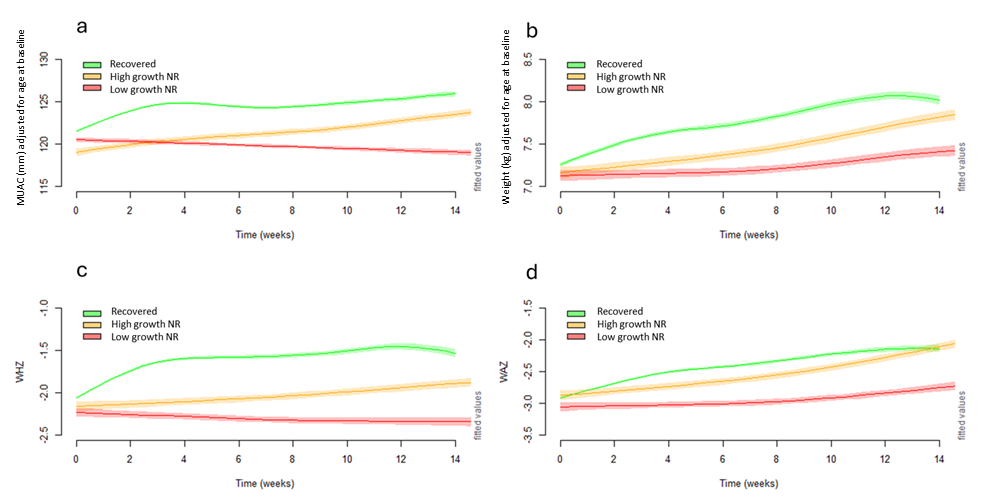

Supplement: S5 Fig — High growth non-responders and low growth non-responders defined using 25th percentile of MUAC gain (panel a, b) and absolute daily weight gain (panel c, d). MUAC, mid-upper-arm circumference; WAZ, weight-for-age z-score; WHZ, weight-for-height z-score. (TIF) [file pgph.0003741.s008.tif]

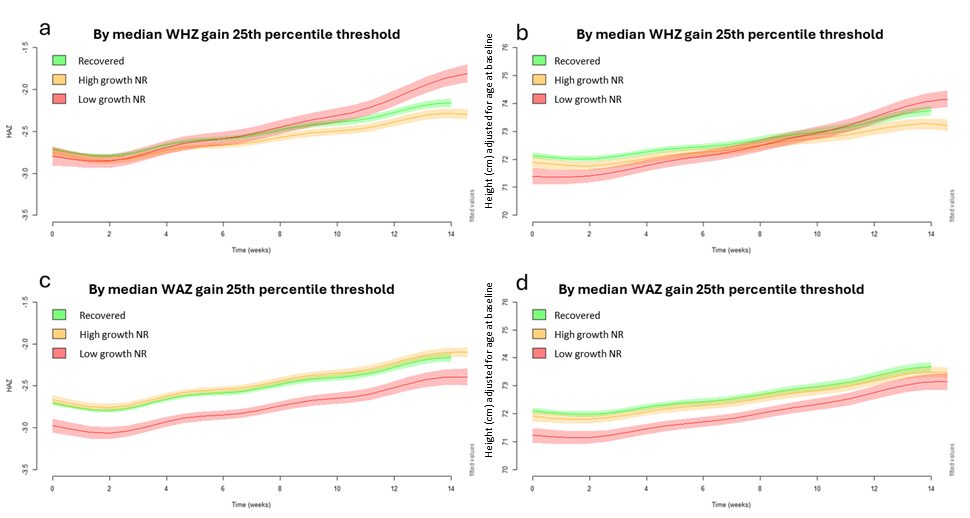

Supplement: S6 Fig — High growth non-responders and low growth non-responders defined using 25th percentile of absolute median WHZ gain (panel a, b) and absolute median WAZ gain (panel c, d). MUAC, mid-upper-arm circumference; WAZ, weight-for-age z-score; WHZ, weight-for-height z-score. (TIF) [file pgph.0003741.s009.tif]
