# Supplementary material for: Re-thinking “non-response” to wasting treatment: Exploratory analysis from 14 studies
Source: PLOS Glob Public Health. 2025 Feb 12;5(2):e0003741. doi: 10.1371/journal.pgph.0003741 (PMC11819528; doi:10.1371/journal.pgph.0003741)
Supplement: Attachment — Submitted filename: Letter responding to peer review comments FINAL.pdf [file pgph.0003741.s011.pdf]

Emergency Nutrition Network
69 High Street
Kidlington
OX5 2DN
UK

www.ennonline.net
office@ennonline.net

30th August 2024

Dear Dr Gonzales,

Re-thinking "non-response" to wasting treatment: exploratory analysis from 14 studies

We would like to thank you and the two reviewers for taking the time to consider our submission, and for the encouraging and considered comments that you all provided.

Please find below our point-by-point response to the comments in red font.

Editor:

After careful consideration, we feel that it has merit but does not fully meet PLOS Global Public Health's publication criteria as it currently stands. Therefore, we invite you to submit a revised version of the manuscript that addresses the points raised during the review process. Additionally, I encourage the authors to strongly consider the comments of Reviewer 1 about the dichotomisation of the growth trajectories.

Thank you for offering the opportunity to submit a revised manuscript. The comments from the reviewers have helped us see that our original methods section detailing the dichotomisation of the growth trajectories was not clear nor comprehensive enough. We are grateful to have had the chance to explain the rationale that underpinned our approach in more detail. We trust our revisions have strengthened the paper with a clearer description of our methods and a more comprehensive acknowledgement of the limitations of our data. We have edited our acknowledgements section to thank the three of you for your role in strengthening our paper. Line numbers in our response below refer to the clean (or tracked changes in simple markup view) Word document.

Reviewer #1:

This thought-provoking paper deals with the important question of how we define non-response in children being treated for SAM/severe malnutrition/wasting (nomenclature in flux). The authors have assembled a dataset of 14 studies and reanalysed their data, representing 22,685 children but excluding 4,631 with oedematous malnutrition. The manuscript is well written and clear.

I have one reservation. The whole analysis rests on a comparison of the growth trajectories dichotomised into groups above and below the 25% centile of anthropometric gain during follow up. The authors conclude that the upper 75% actually grow fairly well, but the bottom 25% don't. Without a sensitivity analysis, showing that other dichotomies do not discriminate so well between good and poor growth trajectories, the authors have merely generated a very sophisticated way of showing that children growing well grow well, and that children growing badly grow badly. I would

like to see evidence that dichotomising around 40% or 15% is less effective at predicting poor recovery. This would also make the ROC analysis less arbitrary. The methodological explanation given in lines 125-127 is not really very helpful, but the authors do acknowledge the difficulty in lines 333-335. A sensitivity analysis would go a long way to resolving this.

Thank you for your comments, which have helped us see that our original methods section regarding the dichotomisation explained what we did, but not why we did it. As such, this made our selection of the 25th percentile seem arbitrary. However, our original decision to use this threshold was based on the data.

In S3 Table we present the characteristics of participants at enrolment, comparing those children who recovered versus those categorised as non-responders. We saw that for absolute MUAC gain the median was very similar between the recovered and non-responder groups, but the interquartile range was different. The IQR for the non-responders was 1mm - 10mm, which showed us a quarter of these children were not growing at all (<1mm growth). Three-quarters of these children were therefore displaying MUAC growth despite being labelled non-responders. We found the same when we looked at the median and IQR of absolute WHZ gain. Again, we have a similar median WHZ gain between the two groups but for the non-responders we had a wide IQR (0.0- 1.3 WHZ) that showed us one-quarter of the children were not growing at all, but three-quarters were. We decided it would therefore be useful to explore splitting the non-responder category into two groups using the 25th percentile.

The difficulty of choosing another threshold to split the groups is that the decision would not be driven by our data in the same way. For example, using the 40th percentile is very close to the median and would result in very similar profiles between the low growth and high growth NR groups. Using the 15th percentile would result in some children who did not grow at all being included in the high growth group. We therefore would prefer to keep to our threshold using the 25th percentile, which we hoped we have now shown was driven by patterns we saw in our data.

It is true that whichever threshold we choose, as you have said, we are showing that some non-responders grow well, and some grow badly. However, we feel this is the crux of our paper – although it seems an obvious observation from our data, these children are currently all thrown into one category within their treatment programmes, whereas we argue that they should be considered as different groups with potentially different programmatic approaches, to be tested in future research.

We have aimed to clarify the above by editing the methods section from line 126 onwards:

"We found that for several anthropometric outcomes (e.g. absolute MUAC gain, absolute WHZ gain) the median values between the two groups were similar although the IQRs differed. Intriguingly, the IQR of the non-responders showed that children in the first quartile were not growing at all (e.g., absolute gain near 0), but that three-quarters of the children were indeed growing. We therefore split the non-responder group into two further categories: low growth non-responders ('low growth NR') and high growth non-responders ('high growth NR')…"

Minor points

- Figure 1 – should be "adjusted for", not "adjusted on".

Thank you, we have corrected all main and supplementary figures accordingly.

- Lines 335-6 – "self-reported morbidities" is surely inappropriate for children. (reported morbidities)

You are correct, this was an error and we have now edited to "reported morbidities", as in some studies this was done by the caregiver and in other settings by the health workers.

- Lines 337-387 – isn't it self-evident that very malnourished children are more likely to take time to respond and have a higher risk of non-response?

We agree with the point when we are considering the originally-categorised non-responder group with recovered children. We do see (in S3 Table) that non-responders start off with worse anthropometric deficits than those who go onto recover, and therefore are likely to take more time to respond. However, what we have aimed to bring out in the discussion is that when we split the non-responders into the 'low growth' and 'high growth' groups, the expected pattern isn't as clear. We expected the low growth NRs to be those having the worst anthropometric status at enrolment, but this was not the case. In Table 1 we see the low growth NRs had an equal or better starting anthropometry compared to the high growth NRs. We have therefore aimed to draw attention in our discussion to the difficulty in predicting which children will end up in which non-responder group.

Reviewer #2:

This is an important topic, a novel approach to thinking about it and is valuable to the SAM treatment community. The analyses appear appropriate. I have only relatively minor comments that may improve the manuscript.

In the Abstract, a couple of things were unclear or ambiguous:

- It would be helpful to indicate whether the 25th percentile gain is with reference to the study populations(s) or WHO standards.

Thank you. In light of Reviewer 1's comment on our methods, and your comment here, we have revised the abstract to clarify our approach. The methods section of the abstract now reads:

"Observing the pooled data of non-responders' mid-upper arm circumference (MUAC), weight, weight-for-age z-score, weight-for-height z-score and daily weight gain rate, we found that the first quartile differentiated those who did not grow at all versus those that demonstrated some growth. We therefore defined 'low growth non-responders' as < 25th percentile anthropometric gain between admission and exit using the non-responders' pooled data, and 'high growth non-responders' as ≥ 25th percentile gain."

- 'For all outcomes, the high growth non-responders tracked along a near-parallel growth trajectory to children who recovered but started with lower anthropometry' – does this mean that the non-responders or those who recovered had lower initial anthropometry?

We have edited the abstract to make this clearer, now reading:

"For all outcomes, the high growth non-responders started with a worse anthropometric status compared to those who recovered, but then tracked along a near-parallel growth trajectory."

- At lines 128-130, there is the same issue of what the percentile refers to.

We have now added in that the percentile refers to the non-responders' pooled data, thank you (now line 135).

- At line 134, 'mixed effects' could mean clustered by individual and/or site – please clarify. Likewise, at line 155, were shared characteristics within sites accounted for?

Thank you, we have now clarified that the mixed effect model accounted for clustering at the individual level (now line 141). The model did not cluster at the site level – we did originally attempt this, but the model could not achieve convergence due to some sites having too small a sample size.

- Might the context of the study be important? For example, endemic malnutrition vs. sudden food insecurity shock may mean different underlying deficiencies and responses of weight, height and MUAC to treatment. Could this be tested, e.g. in results presented in Table 1? At line 302 I was also wondering if this co-variate might help differentiate between the risk of being in the high growth NR or low growth NR groups. Presumably, the community norm might also influence when and at what level trajectories flatten.

Thank you, this is a very good point and one that generated a lot of discussion as we were planning our paper! It is unfortunate that we do not have the necessary contextual information within our included datasets to test this point, but we agree that type of shock and the level of undernutrition in the general population would likely influence the level of non-response we find. For example, in one extreme where we have a population where all children are well-nourished, 2.5% of children will be below -2 z-score (WHZ, WAZ or any other index). If we try to supplement these children, it may be unlikely that they will respond to treatment. On the other hand, if we have a population where all children are malnourished, we might expect most of them to catch up weight after supplementation, even those who are above -2 z-scores. In summary, we might expect the proportion of "non responders" to depend on how far to the left the population distribution of various anthropometric indicators are *i.e.* response to supplementation may indeed depend on the overall population nutritional status (which may also depend on the type of shocks, as you have suggested).

We have added this point in our discussion under the limitation section (now line 497 onwards):

"The included datasets did not include contextual factors such as the nutrition status of the general population, or the type of nutritional shocks being experienced (e.g. endemic malnutrition versus sudden food insecurity shocks). These contextual factors could influence what proportion of children respond to treatment and at what point growth trajectories flatten – considerations that would be interesting to explore in further research".

- At line 279, did age influence the time to cross MUAC 115mm? At age 6 months, infants may struggle more to reach a threshold than a 4 year old since population MUAC is not flat from 6-59 months.

We did not explicitly look at the time to cross MUAC 115mm in our analyses. However, we do agree that age likely plays a role in the growth trajectories. We adjusted for age at enrolment in all our grow curves for this reason. We also explored the effect of age in the multinomial model (fig 6). Compared to children who recovered, children in older age categories had increased odds of being a high growth or low growth NR. However, age did not discriminate between the high growth and low growth NR groups.

- At line 410, non-anthropometric individual risks like low birth weight, breast feeding etc could be mentioned, as they are mentioned as limitations of the datasets at line 432 (perhaps this should be under line 481).

Thank you, we agree. We have added these considerations to our limitations section, now line 499.

Finally, a paper on non-responders from Niger was just published, showing similar results in the proportion of children originally categorised as non-responders who could be better categorised as ‘slow responders’ (corresponding to our high growth NR group) and ‘treatment failures’ (corresponding to our low growth NR group). We therefore took the opportunity to refer to this recent research in our discussion (lines 480-483).

We thank the reviewers again for the time they took to help us improve our manuscript.

Your sincerely,

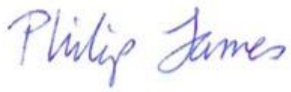

Philip James, PhD

On behalf of the co-authors Cécile Cazes, Heather Stobaugh, Paluku Bahwere, Paul Binns, Robert E. Black, Erin Boyd, André Briend, Sheila Isanaka, Suvi T. Kangas, Tanya Khara, Natasha Lelijveld, Martha Mwangome, Mark Myatt, Gloria Odei Obeng-Amoako, and Indi Trehan.

Philip James, PhD
Senior Technical Associate
Emergency Nutrition Network (ENN)
t: +44 (0) 7971 263776
e: philip@ennonline.net w: www.ennonline.net
