# Supplementary material for: Re-thinking “non-response” to wasting treatment: Exploratory analysis from 14 studies
Source: PLOS Glob Public Health. 2025 Feb 12;5(2):e0003741. doi: 10.1371/journal.pgph.0003741 (PMC11819528; doi:10.1371/journal.pgph.0003741)
Supplement: Attachment — Submitted filename: R2 Letter responding to peer review.pdf [file pgph.0003741.s012.pdf]

Emergency Nutrition Network
69 High Street
Kidlington
OX5 2DN
UK

www.ennonline.net
office@ennonline.net

26th October 2024

Dear Dr. Amugsi,

PGPH-D-24-01377R1: Re-thinking "non-response" to wasting treatment: exploratory analysis from 14 studies

We would like to thank you and the three reviewers for taking the time to consider our revised manuscript. We have updated our acknowledgements section accordingly. Please find below our point-by-point response to the comments in red font. Our line numbers refer to the manuscript in 'simple markup' view.

Reviewer #1: I am disappointed that the authors have elected not to carry out the (not very onerous) additional analysis that I feel is required to permit the conclusions drawn. I cannot support the publication of this manuscript as is.

We thank the reviewer for reading through our revised manuscript. We do appear to have a difference of opinion on the necessity of the requested additional analyses, and respectfully request the following considerations be made as part of the editorial decision.

- The publication criteria for this journal contain 6 key criteria and we believe we have met all of them.
- We note that during the first round of reviews, reviewer 1 answered "YES" to the question of whether our manuscript met the journal's publication criteria. Now, because we have not performed the additional analyses it seems the reviewer has changed the response to "NO". We understand the reviewer is disappointed in not seeing their recommendation followed but feel this does not justify changing the assessment of our manuscript from meeting the publication criteria to now not meeting them.
- Reviewers 2 and 3 have not requested the additional analyses.
- In our original response letter, we provided a detailed justification for using our cut-offs originating from the interquartile range of the non-responders' data, explained that choosing another threshold to split the groups would not be driven by our data, and illustrated some of the limitations of choosing different thresholds.
- We did find the reviewer's original comments very helpful for us to clarify our methods section in our revised manuscript.

Considering the above points, our strong preference would be not to run additional analyses. However, as editor, if you would like to see these additional analyses, please be reassured

that if requested by you we can provide the additional analyses, and you can assess whether they belong in supplementary material or not.

Reviewer #2: My comments have been addressed

Thank you for your time in checking our previous response against your comments.

Reviewer #3: The research question is interesting and important.

We are very grateful to reviewer 3 for her comments and insight, which gave us the opportunity to clarify some of the findings and allow us to enhance and supplement the discussion with important additional insights.

Specific reviewer questions/comments:

1. "Children who were severely underweight on admission had a higher risk of being in the low growth non- response group."

This statement in the manuscript seems contrary to what is shown on Table 1: at baseline, 78% of High Growth Non-Responders were SAM, while 50% of Low Growth Non-Responders were SAM.
Furthermore, supplemental figures show that baseline anthropometry measures of Low Growth Non-Responders were closer to those of Recovered children. Please clarify.

We thank the reviewer for giving the opportunity to clarify this point.

In the univariate analysis in Table 1, we found that children with MAM (compared to those with SAM) had a lower risk of being in both the high growth non-responder (NR) group (OR 0.69 [95% CI: 0.59, 0.80]) and the low growth NR group (OR 0.19 [0.17, 0.22]) compared to the recovered group. This result is consistent with the descriptive results mentioned: 78% of the high growth NRs were children with SAM, while 50% of the low growth NRs had SAM.

The univariate analysis in Table 1 shows that children who were severely underweight had a higher risk of being in both NR groups compared to being in the recovered group (OR 1.64 for the high growth NRs and OR 1.51 for the low growth NRs). However, when we consider the multivariate analysis (shown in Figure 6), we see the severe underweight criteria only remains significant for predicting those in the low growth NR group. This means that after adjusting for sex, age, MUAC, WHZ at admission, MUAC or weight change at least once during the first month, and infectious morbidity during the treatment, children with WAZ<-3 at admission remain more likely to belong to the low-growth NR group (aOR: 1.6 [1.2-2.0]).

Regarding the baseline anthropometry measures of low growth NRs being closer to those of children who recovered, we found slightly different starting points according to the anthropometric indicator and category of acute malnutrition. For example, in Figure 3 (showing all children), children in the recovered and low growth NR responder groups indeed started with a similar MUAC at baseline (adjusted for age). However, the low growth NR group's WHZ and WAZ at baseline, after adjusting for age, was slightly lower than for those who recovered. Then in Figure 4 (growth curves among children with SAM only), MUAC at baseline is slightly higher in the low growth NR group compared to the recovered group. In summary, we aimed to describe the shape of the growth curves over time in the narrative, as

we felt this was where the interesting differences between the curves, with implications for programming, became apparent.

2. Age of child at baseline is not evenly distributed among the three study groups, as shown on Table 1. High Growth Non-Responder children were significantly more likely to be in the 6-11 month-old group as compared to the other two groups at baseline. Low Growth Non-Responder children were much more likely to be in the 24-59 month-old group. A suggestion is to include discussion of how age differences could have affected the trajectories of growth recovery in the three study groups, and how this could be related to gastrointestinal changes that occur as a reaction to wasting, especially when wasting is prolonged before feeding treatment is started.
Did the authors consider a case-control study, which could have adjusted for age and possibly other factors?

We absolutely agree with the reviewer that age at admission is a major characteristic that can influence the growth curves. For this reason, we included age as an adjustment variable in all models of growth for MUAC and weight. For WHZ, the performance tests on growth curves showed better predictive results without adjustment on age at baseline. We considered whether different age categories influenced the likelihood of being in a certain NR group in figure 6. After adjusting for all other variables in the model, we found that older children (compared to the youngest strata of 6-11 months) had a reduced odds of being in both the low growth NR and high growth NR groups compared to children who recovered. It was interesting that the direction of effect sizes by age did not change between the two groups in figure 6. Hence, we feel we did appropriately consider age in our models.

We did not consider a case-control study because our analyses were already adjusted for age, both for growth curves and the multivariate model. However, the point about gastrointestinal changes is well taken, thank you, and we address this in the next comment.

3. It would be important to include discussion of the link between wasting and the status of the gastrointestinal tract (including the possibility of villous blunting, small bowel bacterial overgrowth, exocrine pancreatic insufficiency, among others) and how that affects growth trajectories and response to food-based treatment regimens. Also, how this could be the case especially in the Low Growth Non-Responders who may need further medical assessment and additional non-food related treatment.

We are grateful to the reviewer for raising this important point. We have amended the discussion section accordingly in lines 372-375. (Note to editorial team, two references have been added accordingly).

4. Given that the journal PGPH is oriented to global public health, it would seem useful to include text in the background section on the overall magnitude of SAM and MAM in children in LMICs, and on the general issue of early prevention of moderate and severe acute malnutrition in children.

We agree and have now included this information as recommended in lines 31-35. (Note to the editorial team that this has resulted in 4 new references added).

5. The manuscript includes some suggestions of ways to follow-up the Low Growth Non-Responders. It could appear not useful to the tone of the manuscript to mention cost-

implications of prolonging care of low-growth non-responders which could be interpreted as a deterrent to prolonged care. Discussion of cost could more positively be incorporated as the cost-effectiveness of early prevention, early identification, and early treatment of wasting.

We thank the reviewer for this suggestion and have included this positive view in the Discussion, lines 438-440.

Your sincerely,

Philip James, PhD

On behalf of the co-authors Cécile Cazes, Heather Stobaugh, Paluku Bahwere, Paul Binns, Robert E. Black, Erin Boyd, André Briend, Sheila Isanaka, Suvi T. Kangas, Tanya Khara, Natasha Lelijveld, Martha Mwangome, Mark Myatt, Gloria Odei Obeng-Amoako, and Indi Trehan.

Philip James, PhD
Senior Technical Associate
Emergency Nutrition Network (ENN)
t: +44 (0) 7971 263776
e: philip@ennonline.net w: www.ennonline.net
