# Supplementary material for: Re-thinking “non-response” to wasting treatment: Exploratory analysis from 14 studies
Source: PLOS Glob Public Health. 2025 Feb 12;5(2):e0003741. doi: 10.1371/journal.pgph.0003741 (PMC11819528; doi:10.1371/journal.pgph.0003741)
Supplement: Attachment — Submitted filename: R3 Letter responding to peer review_final.pdf [file pgph.0003741.s013.pdf]

Emergency Nutrition Network
69 High Street
Kidlington
OX5 2DN
UK

www.ennonline.net
office@ennonline.net

6th December 2024

Dear Dr. Amugsi,

PGPH-D-24-01377R2: Re-thinking "non-response" to wasting treatment: exploratory analysis from 14 studies

We would like to thank you and the reviewers for taking the time to consider our revised manuscript. Please find below our point-by-point response to the comments. To maintain the full conversation, we have kept the relevant previous comments and responses in black font, highlighted the new comments in blue font, and provided our new responses in red font. Our line numbers refer to the manuscript in 'simple markup' view.

Additional Editor Comments (if provided):

1. Reviewer #3 notes potential confusion in the manuscript regarding growth categories and their implications on treatment expectations, particularly in the statement about severely underweight children (WAZ <-3). The reviewer highlights that this statement in the abstract sets an expectation for "treatment failures" among children with severe underweight, which may be confusing based on the results presented in Table 1. They recommend an expanded explanation beyond the regression model findings.
2. Additionally, Reviewer #3 suggests that a discussion on age-related differences in outcomes for children starting feeding programs at younger versus older ages, supported by relevant literature, would be valuable.

Thank you for summarising the two reviewer comments for us to address. We provide detailed responses to each below. In addition, we a made minor edit in line 17 to clarify we were looking at predictors of non-response category.

Reviewer 3 specific comments

1. Reviewer comment1: "Children who were severely underweight on admission had a higher risk of being in the low growth non- response group." This statement in the manuscript seems contrary to what is shown on Table 1: at baseline, 78% of High Growth Non-Responders were SAM, while 50% of Low Growth Non-Responders were SAM. Furthermore, supplemental figures show that baseline anthropometry measures of Low Growth Non-Responders were closer to those of Recovered children. Please clarify.

Author response1: We thank the reviewer for giving the opportunity to clarify this point.
In the univariate analysis in Table 1, we found that children with MAM (compared to those with SAM) had a lower risk of being in both the high growth non-responder (NR) group (OR 0.69 [95% CI: 0.59, 0.80]) and the low growth NR group (OR 0.19 [0.17, 0.22]) compared to the recovered group. This result is consistent with the descriptive results mentioned: 78% of the high growth NRs were

children with SAM, while 50% of the low growth NRs had SAM.

The univariate analysis in Table 1 shows that children who were severely underweight had a higher risk of being in both NR groups compared to being in the recovered group (OR 1.64 for the high growth NRs and OR 1.51 for the low growth NRs). However, when we consider the multivariate analysis (shown in Figure 6), we see the severe underweight criteria only remains significant for predicting those in the low growth NR group. This means that after adjusting for sex, age, MUAC, WHZ at admission, MUAC or weight change at least once during the first month, and infectious morbidity during the treatment, children with WAZ<-3 at admission remain more likely to belong to the low-growth NR group (aOR: 1.6 [1.2-2.0]).

Regarding the baseline anthropometry measures of low growth NRs being closer to those of children who recovered, we found slightly different starting points according to the anthropometric indicator and category of acute malnutrition. For example, in Figure 3 (showing all children), children in the recovered and low growth NR responder groups indeed started with a similar MUAC at baseline (adjusted for age). However, the low growth NR group's WHZ and WAZ at baseline, after adjusting for age, was slightly lower than for those who recovered. Then in Figure 4 (growth curves among children with SAM only), MUAC at baseline is slightly higher in the low growth NR group compared to the recovered group. In summary, we aimed to describe the shape of the growth curves over time in the narrative, as we felt this was where the interesting differences between the curves, with implications for programming, became apparent.

Reviewer response 2: Thank you for this explanation. However, the statement remains confusing as most children in the 3 growth categories were severely underweight (WAZ<-3). This statement in the abstract that I asked about is important since it leads the reader to expect that the children with severe underweight (i.e. WAZ<-3) at baseline will be those most at risk of being "treatment failures" as you call them, and sets up expectations for such to occur for those children. The confusion is that Table 1 shows that, compared to Low Growth NR and Recovereds, High Growth NR has at baseline: highest percentage of WAZ <-3; lowest mean WAZ; lowest weight; highest percentage SAM; and highest % MUAC <115 cm. The multiple regression model indeed shows that LG-NR has significantly higher OR of WAZ <-3 versus the recovered. This should be explained beyond what the regression shows.

Thank you for your comments. We agree with you that by singling out the severely underweight predictor in the abstract we risk over-simplifying the findings from our multivariate analyses (fig 6) and causing potential confusion.

Therefore, in the abstract we have now:

a) Clarified that the statement on predictors comes from multivariate analyses
b) Emphasised that the comparison group is the recovered children
c) Included all three co-variates that differentiated the odds of being in the high growth or low growth non-responder groups: WAZ <-3, MUAC 115-119 and sex of child.

Our amended abstract now reads (lines 20-23): "In multivariate analyses, children who were severely underweight or had MUAC 115-119mm on admission had higher odds of being in the low growth non-response group compared to being recovered. Boys had reduced odds of being in the high growth non-response group than girls".

Where we mention severe underweight as a predictor in the discussion, we have now:

- Reminded readers the finding comes from multivariate analyses in line 371
- Added additional context in lines 398-403 where we talk about the difficulty of predicting which children will end up in which groups:

> "Severe underweight on admission was associated with increased odds of being in both the low growth NR and high growth NR groups compared to recovered children in univariate analyses (Table 1), whilst in multivariate analyses (fig 6) severe underweight was only associated with increased odds of being in the low growth NR group. Therefore, whilst this indicator alone is insufficient to fully predict outcomes, it does mean health workers should be aware of the increased vulnerability of children who are severely underweight at admission".

We hope that the above changes have now better reflected the context of our findings related to severe underweight on admission.

2. Reviewer comment1: Age of child at baseline is not evenly distributed among the three study groups, as shown on Table 1. High Growth Non-Responder children were significantly more likely to be in the 6- 11 month-old group as compared to the other two groups at baseline. Low Growth Non-Responder children were much more likely to be in the 24-59 month-old group. A suggestion is to include discussion of how age differences could have affected the trajectories of growth recovery in the three study groups, and how this could be related to gastrointestinal changes that occur as a reaction to wasting, especially when wasting is prolonged before feeding treatment is started.
Did the authors consider a case-control study, which could have adjusted for age and possibly other factors?

Author response: We absolutely agree with the reviewer that age at admission is a major characteristic that can influence the growth curves. For this reason, we included age as an adjustment variable in all models of growth for MUAC and weight. For WHZ, the performance tests on growth curves showed better predictive results without adjustment on age at baseline. We considered whether different age categories influenced the likelihood of being in a certain NR group in figure 6. After adjusting for all other variables in the model, we found that older children (compared to the youngest strata of 6-11 months) had a reduced odds of being in both the low growth NR and high growth NR groups compared to children who recovered. It was interesting that the direction of effect sizes by age did not change between the two groups in figure 6. Hence, we feel we did appropriately consider age in our models.

We did not consider a case-control study because our analyses were already adjusted for age, both for growth curves and the multivariate model. However, the point about gastrointestinal changes is well taken, thank you, and we address this in the next comment.

Reviewer response2: Thank you for this explanation. However, it would be useful to include more discussion on the differences that could be expected between children who begin feeding programs at a younger age versus an older age, and why. No doubt there is literature available that could be cited.

Thank you for your comment. Indeed, in our multivariate analysis (figure 6), compared to those who recovered, older children had a reduced odds of being in both the high growth NR and low growth NR groups. Whilst age therefore did not differentiate the risk of being in a certain NR group, your comment helped us see we had missed out discussing age altogether when explaining the results of figure 6. We had listed all the co-variates that had increased the odds of being in both NR groups, but not then listed the co-variates that decreased the odds of being in both groups. We have now amended this in our results. Line 320-22 now reads: "Compared to the youngest age bracket (6-11 months), older children had reduced odds of being in both the high growth NR and low growth NR groups compared to recovered children "

We have further explored this point later in the discussion by adding in the following (lines 404-410; note to editor we have added a reference accordingly):

"Various co-variates increased or decreased the odds of being in both the high growth and low growth NR groups, but did not differentiate between the two NR groups. For example, older children (age 12-59 months) had reduced odds of being in both NR groups compared to younger children (age 6-23 months). This is consistent with other research showing that younger children are more at risk of being non-responders (40), and may in part be because in MUAC-based programming younger children tend to have a smaller MUAC on admission than older children and take longer to reach the discharge criteria".

Thank you for your support in improving the manuscript.

Your sincerely,

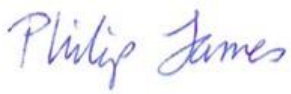

Philip James, PhD

On behalf of the co-authors Cécile Cazes, Heather Stobaugh, Paluku Bahwere, Paul Binns, Robert E. Black, Erin Boyd, André Briend, Sheila Isanaka, Suvi T. Kangas, Tanya Khara, Natasha Lelijveld, Martha Mwangome, Mark Myatt, Gloria Odei Obeng-Amoako, and Indi Trehan.

Philip James, PhD
Senior Technical Associate
Emergency Nutrition Network (ENN)
t: +44 (0) 7971 263776
e: philip@ennonline.net w: www.ennonline.net
